# The Effects of Urbanization on the Infant Gut Microbiota and Health Outcomes

**DOI:** 10.3389/fped.2020.00408

**Published:** 2020-07-29

**Authors:** Siobhan Brushett, Trishla Sinha, Sijmen A. Reijneveld, Marlou L. A. de Kroon, Alexandra Zhernakova

**Affiliations:** ^1^Department of Genetics, University Medical Center Groningen, University of Groningen, Groningen, Netherlands; ^2^Department of Health Sciences, University Medical Center Groningen, University of Groningen, Groningen, Netherlands

**Keywords:** urbanization, gut microbiota, infant, mother, health outcomes, delivery mode, nutrition, medication

## Abstract

Humans and their gut microbiota have co-evolved over thousands of years, resulting in the establishment of a complex host–microbiota ecosystem. Early life environmental factors, such as delivery mode, nutrition, and medication use, have been shown to substantially affect both host–microbiota interactions and health outcomes. However, the effects of urbanization (characterized by the spectrum of rural and urban populations) on these early life events have been overlooked. A deeper understanding of the relationship between urbanization and microbiota development will allow for the identification of novel biological and social approaches that can be implemented to prevent and treat disease and promote maternal and infant/child health. The aim of this narrative review is to summarize how factors associated with urbanization differentially impact delivery mode, nutrition, and medication use, and how these changes subsequently affect the gut microbiota and health outcomes of infants. This narrative review also describes the important evidence gaps associated with these relationships and recommends actions that can be taken to improve the health of mothers and infants worldwide.

## Introduction

Of the many relationships that humans experience in their lifetime, the relationship between human host and gut microbiota is possibly one of the most important. This relationship has developed over thousands of years, allowing for the establishment of longstanding and essential bi-directional host–microbiota interactions. Host–microbiota interactions and functions influence nutrient metabolism, vitamin synthesis, mucosal maturation, immune-system development, and biochemical signaling between the gut and the brain (1–4). Accordingly, various human diseases, including immune-related diseases, chronic metabolic diseases, and neurodevelopmental diseases, have been associated with disruptions to the gut microbiota composition and function. Combined, these interactions and associations provide evidence for the important influence of the gut microbiota on human health outcomes.

One important player in this complex host–microbiota relationship is the external environment. Multiple, often interrelated, environmental factors influence both the human gut microbiota and health outcomes. Humans are most susceptible to environmental factors during early life, and their effects on human health can be evident even in later-life. The first 1,000 days of life is a crucial period for the rapid overall growth and development of a child (5), and it is estimated that over 1 million new neural connections are formed every second in the first few years of life (6). In parallel with infant growth and development, the infant gut microbiota is also being established. During the first few months of life the infant gut microbiota is highly dynamic, which is essential for its maturation and for the stimulation of the immune system. Delivery mode, nutrition, and medication use are well-studied environmental factors known to influence the infant gut microbiota and to contribute to early and later health outcomes (7–10). Other factors, such as stress and home environment (e.g., number of siblings, pets), can also play a role (11–13). However, how urbanization may influence these early life relationships has been overlooked [Box 1; (16, 17)].

Box 1 **Urbanization: definition and its association with income group.**

According to the United Nations (UN) Population Division, urbanization can be characterized by the transition from a rural to an urban population. This transition is associated with “explosive growth of cities, and shifts from an agriculture-based economy to mass industry, technology and services” (14). Based on the 2018 Revision of World Urbanization Prospects (produced by the UN), 55% of the world's population lives in urban areas. In 2018, regions exhibiting high percentages of urbanization included Northern America (82%), Latin America and the Caribbean (81%), Europe (74%), and Oceania (68%). Africa, on the other hand, was considered rural, with only 43% of the population living in urban areas (14). These findings demonstrate the general relationship between urbanization and geographical location. Urbanization gradients are also closely linked with income-group (as determined by the World Bank) (14, 15). Most high-income countries, such as Australia, Canada, Japan, the United States of America, and most European countries, exhibit relatively high levels of urbanization (14).

Urban and rural populations can be defined by the different social structures and associated environmental factors that they are exposed to. For example urban and rural populations can differ by demographics, governance, medical interventions, nutrition, hygiene and sanitation, medication use, and cultural beliefs (Box 2). Accordingly, these differences could differentially impact the factors known to shape the infant gut microbiota and related health outcomes. In fact, research in adult populations has already demonstrated that urban diets, increased sanitation and antibiotic exposure contribute to what has been termed “microbiota insufficiency syndrome” (“the loss of microbial taxa and associated [microbial] functions that were part of our evolutionary past”) (4, 18). Adult microbiota composition can even differ across urbanization gradients within the same country (19). Combined, these factors have also been associated with an increase in immune and metabolic diseases in urban adult populations (4).

Box 2 **Differences between urban and rural settings.**

**Table:** 

Demographics	Age distribution, annual income, social class, population density, general health (incl. non-communicable diseases, communicable diseases, co-morbidities, maternal, and infant mortality).
Governance	Policies and regulatory systems, services such as education and healthcare (incl. accessibility, availability, and affordability).
Medical interventions	Cesarean section delivery rates.
Nutrition	Food environmentts (accessibility, availability, and affordability), industrialized and/or processed foods (incl. antibiotic use in food production), low fiber/high fat diets, high fiber/low fat diets, etc.
Hygiene and sanitation	Access to clean and running water, sewage disposal, plumbing, food, domestic, and public hygiene.
Medication use	Antibiotic use (incl. prophylactic antibiotic administration during cesarean section and overuse/misuse of antibiotics), traditional and complementary medication use, further medication use associated with disease.
Cultural beliefs	Westernized and traditional beliefs and practices.

**Figure 1:**
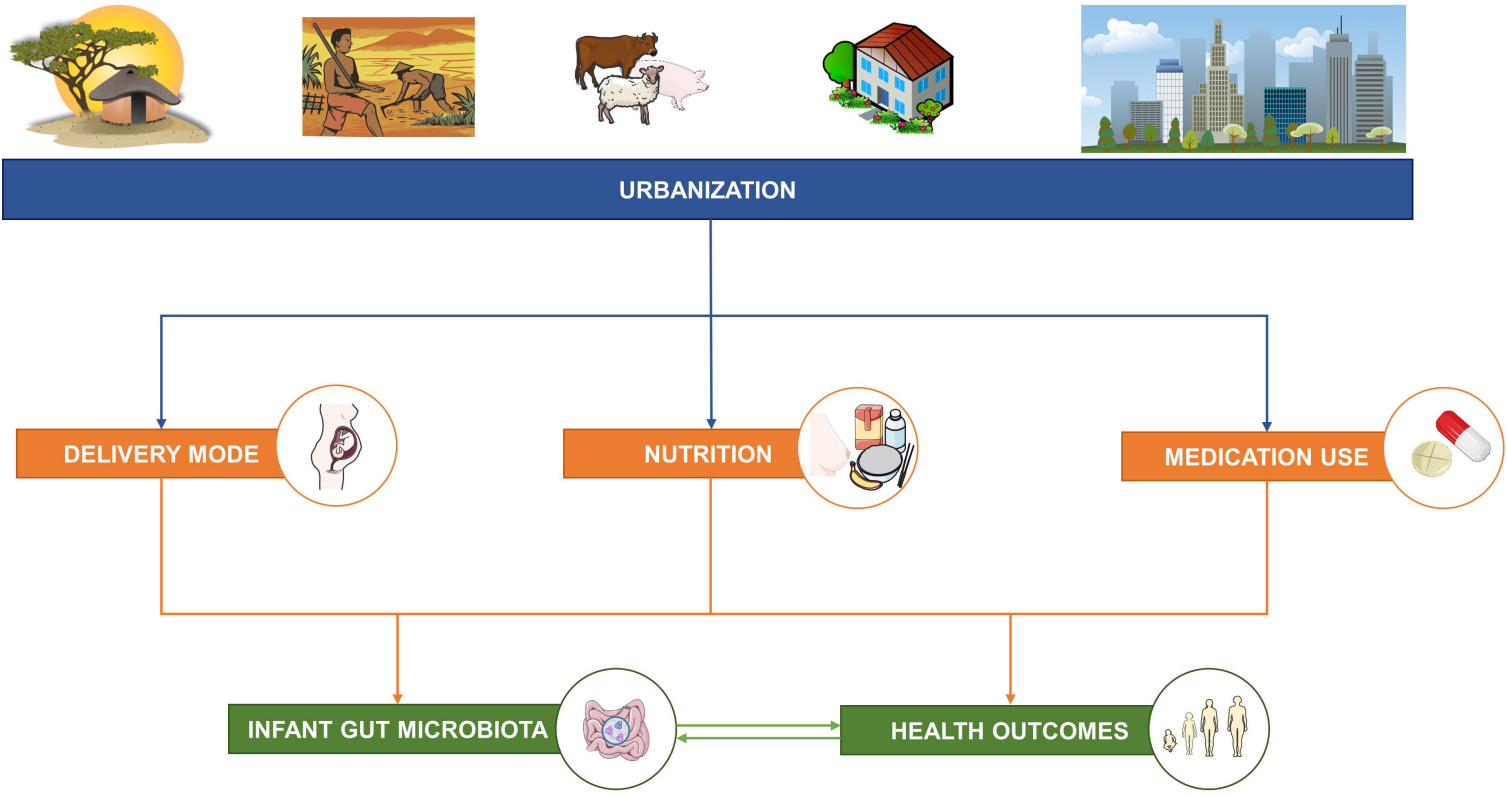
The aim of this narrative review is to describe the relationships between factors associated with urbanization and three well-studied factors—delivery mode, nutrition, and medication use—known to influence the infant gut microbiota and short- and long-term health outcomes. This figure was created using images from Servier Medical Art Commons Attribution 3.0 Unported License (http://smart.servier.com). Servier Medical Art by Servier is licensed under a Creative Commons Attribution 3.0 Unported License.

The aim of this narrative review is to summarize the factors associated with urbanization that differentially impact the well-studied factors of delivery mode, nutrition and medication use, which in turn impact the infant gut microbiota and health outcomes in early and later life (Figure 1; Supplementary Table 1). Next, this narrative review highlights the most important evidence gaps associated with these well-studied factors, such as the relationships with malnutrition in all its forms. This understanding provides insights for the potential use of biological applications (e.g., modification of the gut microbiota by diet) and the need for standardization of certain mother–child health policies across urbanization gradients, which will contribute to efforts to improve the health outcomes for mothers and their infants/children worldwide.

## Delivery Mode, the Gut Microbiota, and Health Outcomes

### The Effects of Delivery Mode on the Gut Microbiota and Health Outcomes

An infant's first encounter with microbes is currently still debated. While some studies provide evidence for *in utero* colonization due to the presence of low abundant microbes in the placenta and amniotic fluid (20–22), others have argued that contamination from sampling and DNA extraction kits cannot be confidently dismissed (23, 24). Regardless, differences between the gut microbiota of infants born by vaginal delivery or cesarean section (C-section) are already evident very early in life. Initially, infants born by vaginal delivery were thought to be exposed to and colonized by the resident microbes of the lower genital tract of the mother, with *Lactobacillus* species being the most dominant (25, 26). However, two recent studies of the neonate gut microbiota showed that the prevalence and abundance of *Lactobacillus* species were similar in both vaginally delivered and C-section groups (27, 28). Instead, *Bifidobacterium, Escherichia, Bacteroides*, and *Parabacteroides* species (Table 1) were enriched in the gut microbiota of vaginally delivered infants, and were possibly inherited from maternal fecal microbes (27, 28). These findings complement those from other studies that demonstrated that maternal gut microbes can be transmitted to infants via vaginal delivery (35, 36). Transmission and colonization of the maternal gut microbiota in infants delivered vaginally is thought to have protective and advantageous functions, as these microbiota are suited to the environment of the gut and have the capacity to contribute to nutrient acquisition in early life (28).

**Table 1:** The influence of delivery mode, nutrition, and medication use on the infant gut microbiota.

**Factor**		**Source of gut microbiota**	**Influence on gut microbiota**	**References**
Delivery mode	Vaginal delivery	Vaginal Canal	Higher abundance of *Lactobacillus* spp.	(7, 26)
		Maternal Gut	Higher abundance of *Bifidobacterium longum* and *B. breve, Escherichia coli, Bacteroides vulgatus*, and *Parabacteroides distasonis*	(27, 28)
	Cesarean section	Skin	Higher abundance of *Staphylococcus, Propionibacterium, Corynebacterium*, and *Streptococcus*	(3, 7, 8)
		Delivery environment	Low initial abundance of *Bacteroidetes* and higher relative abundance of opportunistic pathogens	(27, 29, 30)
Nutrition	Breast milk	Skin and breast milk	Higher abundance of *Streptococci, Staphylococci, Corynebacteria, Lactobacilli, Micrococci, Propionibacteria*, and *Bifidobacteria*	(7, 31–33)
	Formula milk	Gut and environmental bacteria	Higher abundance of *Enterobacteria, Streptococcus, Bacteroides*, and *Clostridium*, as well as members of the genus *Bifidobacterium*	(7, 31)
Medication use	Antibiotic use	–	General decrease in diversity and increase in bacteria containing antimicrobial resistant genes. Reduction in *Clostridiales*, including *Lachnospiraceae*	(9, 34)

In contrast to vaginally delivered infants, infants delivered by C-section are initially colonized by microbes present in the delivery environment, in the maternal mouth and on the maternal skin [Table 1; (3, 7, 8, 25)]. Infants delivered by C-section have a relatively low initial abundance of *Bacteroidetes* (due to inefficient colonization of the gut) and a higher relative abundance of opportunistic pathogens in comparison to vaginally delivered infants (27–29). The effects of C-section delivery on the gut microbiota have been shown to impact the immune and metabolic development of an infant (7, 37, 38). Indeed, increased childhood immune and metabolic diseases have been associated with C-section delivery (38). C-section delivery has also been shown to influence breast milk composition, as mothers who deliver by elective C-section do not release hormones usually associated with labor (39, 40). This suggests a possible role for delivery mode in early life nutrition. Lastly, some infants delivered by C-section are exposed to antibiotics at birth, depending on placental clamping procedures used during prophylactic antibiotic administration (41, 42); the effects of antibiotics on the gut microbiota and health outcomes of infants are covered in the “Medication Use, the Gut Microbiota, and Health Outcomes” section of this review.

### The Effects of Urbanization on Delivery Mode

Although C-section deliveries are an important medical intervention for high-risk pregnancies globally, they are generally over-utilized in low risk pregnancies, particularly in urban and concurrent middle– and high–income countries [Boxes 1, 2; (43)]. According to a WHO report, in 2008, an estimated 6.2 million of the C-section deliveries performed worldwide were unnecessary, with some of the highest C-section rates belonging to China, Brazil and the USA (44).

**Figure 2:**
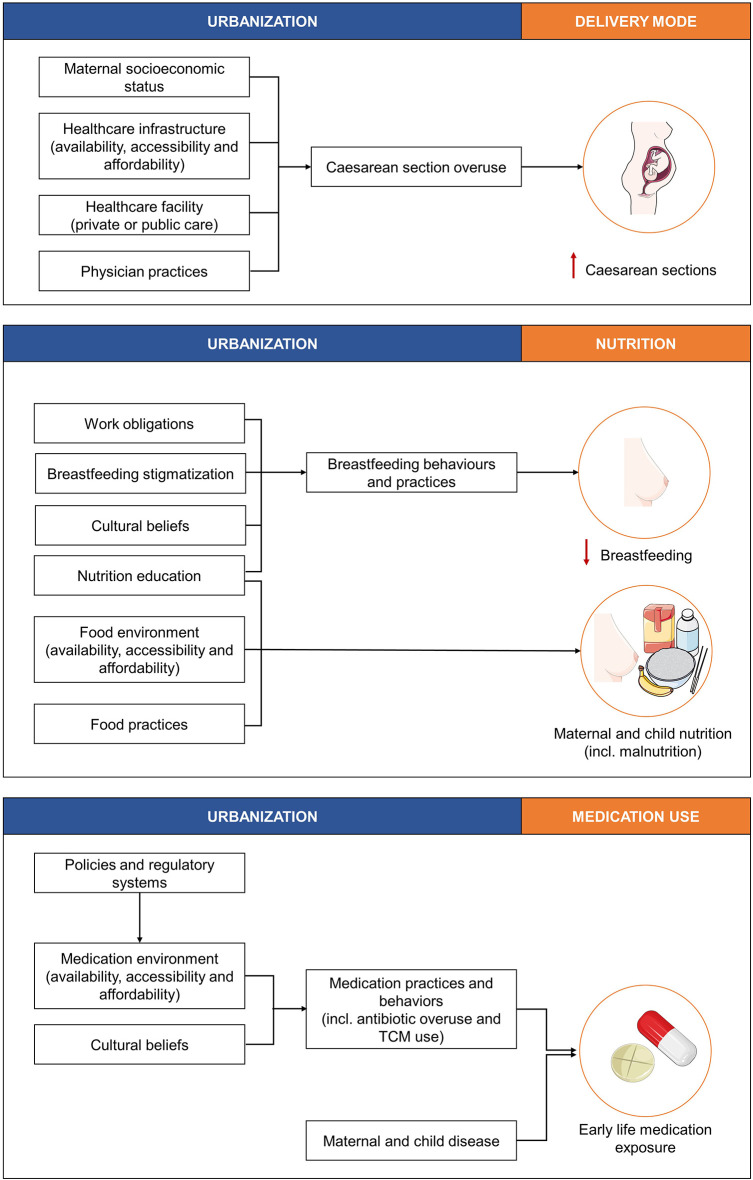
The effects of urbanization on the three well-studied factors, delivery mode, nutrition, and medication use known to influence the infant gut microbiota and short- and long-term health outcomes. TCM, traditional and complementary medication. This figure was created using images from Servier Medical Art Commons Attribution 3.0 Unported License (http://smart.servier.com). Servier Medical Art by Servier is licensed under a Creative Commons Attribution 3.0 Unported License.

Maternal socioeconomic status, healthcare infrastructure (i.e., the accessibility, availability, and affordability of high quality healthcare), type of healthcare facility (i.e., public or private) and physician practices are all factors associated with urbanization that contribute to C-section overuse [Figure 2; (45–47)]. In Brazil, for example the incidence of C-section deliveries was shown to be 1.6 times higher in private facilities compared to public facilities, even though maternal preference was for vaginal delivery (45). Moreover, the effects of physician's practices on C-section overuse can be related to several concerns. This can include lower risks of accusations of malpractice in the event of C-section delivery complications, or time and infrastructure concerns, as C-section deliveries can be “scheduled,” thereby allowing for the time and infrastructure to perform more deliveries (48).

## Nutrition, the Gut Microbiota, and Health Outcomes

### The Effects of Nutrition on the Gut Microbiota and Health Outcomes

Early life nutrition is a well-studied factor that profoundly impacts the infant gut microbiota and is vital for the overall growth and development of a child (5, 49, 50). Feeding modes, such as breastfeeding, formula feeding, mixed feeding, and later weaning (~6 months) have different effects on the gut microbiota and on child health outcomes.

Breastfed infants are exposed to various microbial communities and biological components present on the mother's skin and in breast milk [Table 1; (7, 31, 51)]. These factors are essential for the development of the infant gut microbiota and immune system. This is mainly because the biological components present in breast milk include immune factors (such as antibodies, cytokines, and leukocytes), growth factors and human milk oligosaccharides (HMOs) (52–54). HMOs, in particular, are exclusively found in breast milk and have several beneficial properties. HMOs can: (1) act as metabolic substrates for the growth of beneficial commensal gut bacteria (e.g., *Bifidobacterium*), as HMOs are indigestible by infants, (2) protect against pathogens and toxins by directly binding to pathogens or inhibiting binding to host cell receptors, and (3) contribute to the development of the infant brain (52, 54, 55). Other breast milk components, such as the hormone leptin, are involved in infant appetite regulation, and long-chain polyunsaturated fatty acids facilitate brain development and are protective against type-2 diabetes (T2D) (56).

In comparison to breast milk, formula milk is generally higher in protein content, does not contain leptin, and often does not contain long-chain polyunsaturated fatty acids (56, 57). Some new generation formulas are supplemented with microbiota such as *Bifidobacterium* and *Lactobacillus* species (58). However, due to a lack of data, these supplementations show varying effects (58). Regardless, in general, formula-fed infants show increased gut permeability and bacterial loads, especially of pro-inflammatory taxa of the gut microbiota [Table 1; (57)]. In addition, formula-fed infants exhibit adult-like gut microbiota patterns earlier in life than breastfed infants (7).

Accordingly, breastfeeding is positively associated with child health outcomes. In early life, breastfed infants are less susceptible to infections (such as necrotizing enterocolitis) and diarrhea (56, 59). Later in life, breastfed infants have lower adolescent obesity and T2D risks and better cognitive skills (7, 56, 57, 59–61). In addition, mother–infant bonding during breastfeeding is considered to positively impact infant cognition (56). However, early cessation of breastfeeding, either via the initiation of formula feeding or the premature introduction of solid foods, has been associated with childhood obesity, and facilities the transition of the infant gut microbiota toward an adult-like pattern (5, 7, 62).

Although the implications of breastfeeding and formula feeding have been extensively studied, little information exists on mixed-feeding practices. While this is surprising considering that the majority of infants from middle– and high–income countries are mixed-fed by 3 months of age (57), it is also expected given that the term “mixed-feeding” is not well-defined. Mixed feeding could refer to infants who receive a combination of breast milk and formula milk, or to infants who are initially exclusively breastfed and then switched to formula milk. Only by strictly defining the term “mixed-feeding” can we begin to establish the impacts of early “combined” or “switched” feeding methods on both the gut microbiota and the health outcomes of infants (57).

Following breast-, formula-, or mixed-feeding, infants and children should have adequate access to high quality and diverse foods for optimal growth and development (59). However, when maternal and child nutrition is poor, mothers and their infants can be burdened with malnutrition. Historically, malnutrition was defined by overweight (including obesity) and undernutrition (including stunting and wasting), but a recent series by UNICEF added a third factor termed “hidden hunger” to this definition (59). Hidden hunger refers to micronutrient deficiencies (i.e., lack of vitamins and other essential nutrients) in the diet, which can also contribute to negative health outcomes (59). The effects of malnutrition on the gut microbiota and health outcomes of infants, together with its association to urbanization is described in the “The Effects of Urbanization on Maternal and Child Malnutrition” section of this review.

### The Effects of Urbanization on Nutrition

#### The Effects of Urbanization on Breastfeeding Practices and Behaviors

Nutrition education, cultural beliefs, workforce obligations, and breastfeeding stigmatization are factors associated with urbanization that contribute to the early cessation of breastfeeding, and these factors are differentially observed across urbanization gradients (Figure 2).

Lack of nutrition education differentially affects mothers residing in (and raised in) urban and rural communities. In urban settings, mothers can be inappropriately advised by maternity hospitals to give supplemental infant formula to their infants (57). A survey by the CDC, for example concluded that most hospitals in the USA “do not conform to international recommendations for best practices in maternity care and interfere with mothers' abilities to breastfeed” (63). Across urbanization gradients, early cessation of breastfeeding can be associated with lack of nutrition education and cultural beliefs. In some lessurban Sub-Saharan African countries, for example, breast milk is believed to be lacking in water. Water, which is often contaminated in these settings, and solid foods native to the region are then substituted for breast milk (59, 64). A similar trend can be observed in countries considered to be more urban. Chinese immigrant mothers living in Australia, for example, tend to feed their babies formula milk in the evening, given the recommendation from their elders that doing so facilitates breast milk production because of maternal rest (65). In addition, prior to the recommended weaning age, these mothers also tend to feed their babies water, honey, and rice porridge under the assumption that doing so provides the infant with additional nutrients (65, 66).

Workforce obligations across urbanization gradients significantly contribute to breastfeeding cessation. While mothers (and in some cases fathers) from most countries in the world are entitled to paid parental leave, there are still some countries, such as the United States, that do not guarantee paid parental leave (OECD Family Database). Therefore, along with the effects of an, on average, 18-week period of paid parental leave (in OECD countries), unpaid parental leave places an additional pressure on mothers to return to work early and possibly stop breastfeeding. Indeed, short maternity leave (<6 weeks) has been shown to negatively affect both initiation and continuation of breastfeeding (62). Moreover, regardless of these policies, in rural settings factors such as poverty, income dependency on work and long working hours cause mothers to return to work soon after infant delivery, making it challenging for them to continue breastfeeding exclusively (59).

Breastfeeding stigmatization also plays a role in breastfeeding cessation (67). Mothers have expressed the challenge and stigmatization they experience related to pumping milk in the work place and in relation to public breastfeeding, although the latter is less evident in rural communities (62, 67, 68).

Lastly, it is important to note that numerous other factors contribute to breastfeeding cessation. These can include infant fussiness, low infant weight, pre-term birth and maternal stress and depression (which has also been shown to affect the gut microbiota and health outcomes of infants); all of which, to some degree, could also be associated with urbanization (12, 62).

#### The Effects of Urbanization on Maternal and Child Nutrition

Factors associated with urbanization that affect maternal and child nutrition include food environments (i.e., the accessibility, availability, and affordability of healthy foods), food practices (i.e., food preparation and dietary/lifestyle habits), and nutrition education [Figure 2; (59)]. Maternal and child nutrition further impacts maternal breast milk, and maternal and infant gut microbiota, and the health outcomes of infants.

Geographical location is closely linked with urbanization and the factors associated with it (Boxes 1, 2). Geographical location has been shown to differentially impact breast milk lipids and microbiota composition in mothers from Spain, Finland, South Africa, and China (69). Similarly the breast milk metabolites of mothers from Australia, Japan, the USA, Norway, and South Africa have also been shown to differ (70). Breast milk microbiota composition can also differ across urbanization gradients within countries. For example the breast milk microbiota of mothers residing in a rural village in India showed higher microbial alpha diversity and richness compared to that of Indian mothers residing in an urban metropolitan city (71). The breast milk of the rural mothers also had a moderate abundance of bacteria belonging to the Firmicutes phyla, while the breast milk of the urban mothers exhibited a higher abundance of Proteobacteria (which generally comprise of a variety of pathogenic bacteria) (71). Accordingly, infants from different geographical areas across differing urbanization gradients are exposed to different components in breast milk, which, in turn, could affect the gut microbiota and health outcomes of these infants.

Maternal diet during pregnancy, which differs across urbanization gradient, can also impact the gut microbiota and health outcomes of infants. Though studies of these relationships are limited, a recent *in vivo* mice study has shown that the maternal gut microbiota, in response to different diets during pregnancy, can influence the metabolic outcomes of the offspring (72). This relationship is thought to be mediated by differences in short chain fatty acid production, which bind to receptors in the sympathetic nervous system, adipose tissues, pancreas, and intestine of the embryo (72). Similarly, studies in humans have also demonstrated a relationship between the maternal diet during pregnancy and the infant gut microbiota. First, a study by Lundgren et al. demonstrated that the maternal diet during pregnancy influenced the infant gut microbiota in a delivery mode-dependent manner (73). A second study showed that the maternal diet during pregnancy modulated the maternal gut microbiota, where maternal carbohydrate and protein intake was associated with clusters enriched by *Prevotella*, and maternal fiber, vegetable, protein, and polyphenol intake was associated with clusters enriched by *Ruminococcus*. These differences were further associated with the initial establishment of the infant gut microbiota potentially affecting later life BMI (74).

Urbanization, and the factors associated with it, also differentially impacts the diets of children, thereby differentially affecting the gut microbiota and health outcomes of children. For example the diets of children from a rural village in Burkina Faso were found to be low in fat and animal protein, but rich in starch, fiber, and plant polysaccharides, while the diets of urban Italian children were high in animal protein, sugar, starch, and fat, but low in fiber (75). When comparing the gut microbiota of these two groups, the children in Burkina Faso had a significantly higher abundance of *Bacteroidetes*, but a low abundance of *Firmicutes*, whereas the opposite was observed for Italian children. Notably, higher ratios of *Firmicutes* to *Bacteroidetes* have been associated with a predisposition to obesity (76). It has been shown that an urban diet, especially diets high in fructose and sugar substitutes, can alter the metabolic capacity of the gut microbiota, thereby increasing the risk of obesity and obesity-related diseases [e.g., non-alcoholic fatty liver disease and its more severe form, non-alcoholic steatohepatitis; reviewed in (77, 78)]. In contrast, children in Burkina Faso had significantly more short chain fatty acids than Italian children, and their gut contained a distinct abundance of bacteria (*Prevotella* and *Xylanibacter* genus) associated with maximizing energy intake from fibers in response to a polysaccharide-rich diet, thereby possibly protecting them from inflammation and non-infectious colonic diseases.

#### The Effects of Urbanization on Maternal and Child Malnutrition

Similar to maternal and child nutrition, food environments (i.e., the accessibility, availability, and affordability of healthy foods), food practices (i.e., food preparation and dietary/lifestyle habits) and nutrition education [Figure 2; (59)] are factors associated with urbanization that collectively influence maternal and child malnutrition, and the gut microbiota, and health outcomes of infants and children.

Unsurprisingly, malnutrition in all its forms varies across urbanization gradients and associated income groups (Box 1). For example, overweight is more prevalent in urban communities, yet it is also on the rise in low– and middle–income countries. As a result, individuals from low– and middle–income countries, which struggle with undernutrition, experience a double burden of both overweight and undernutrition (59, 79). Hidden hunger, on the other hand, is a global issue that has been associated with different factors. For example hidden hunger in urban areas is associated with easy access to unhealthy food options (e.g., nutrient poor, ultra-processed, high fat/sugar foods), while in rural areas hidden hunger is associated with the community's dependence on food security (characterized by sufficient access to high quality, nutritious, and diverse foods required to meet recommended dietary needs). Food security in rural areas can be further threatened by climate and seasonal changes (59).

Maternal pre-pregnancy overweight increases the risk of childhood overweight, and has been shown to be mediated by the combined relationship of C-section delivery and *Firmicutes* richness of the infant gut microbiota (80). Maternal overweight can also differentially impact the gut microbiota and health outcomes of infants via breast milk composition. For example breast milk of overweight mothers can differ in comparison to that of normal weight mothers in two ways. First, breast milk of overweight mothers can contain lower levels of transforming growth factor and secretory CD14, which are components that contribute to infant immune system maturation and are initially obtained in low amounts during breastfeeding (81). Second, breast milk of overweight mothers have higher levels of *Staphylococci* (skin bacteria) and lower levels of *Bifidobacteria*, which are important primary colonizers of the infant gut (81). These differences have been generally associated with allergies, inflammatory diseases, and metabolic diseases (81–83). Moreover, maternal overweight during pregnancy is also a predictor of childhood obesity and chronic disease, and can be a risk factor for mothers themselves through increased risks of gestational diabetes, pre-eclampsia and obstetric complications (59, 84).

Along with the effects of maternal overweight, childhood overweight can also impact the gut microbiota and health outcomes of children. Similar to the effects of maternal overweight on the breast milk microbiota, the gut microbiota of overweight children show higher numbers of *Staphylococcus aureus* and lower numbers of *Bifidobacteria* in comparison to normal weight children (82). Moreover, a study by Payne et al. (85) showed that the metabolic activity of the gut microbiota of obese children was increased in comparison to that of normal weight children. Several negative short- and long-term health outcomes have been associated with childhood obesity, including cardiovascular problems, poor self-esteem, and obesity and diabetes in later life (59).

Of the few studies that have been performed on the relationship between maternal undernutrition and the maternal and infant microbiota, most have been performed *in vivo*. For example a mouse study by Connor et al. (86) showed no change in bacterial composition and richness in the gut microbiota of caloric-restricted pregnant mice in comparison to healthy controls. This study, however, was limited by its small sample size and because dams were killed prior to the delivery of the pups, which meant that potential changes to breast milk composition (including, microbiota and biological constituents) and the transmission of maternal gut microbiota to offspring could not be studied (86). The importance of this is demonstrated in a study by Charbonneau et al. (87), which showed that sialylated HMOs were significantly less abundant in the breast milk of Malawian mothers of undernourished infants than they were in mothers of healthy infants. Using two *in vivo* models (in mice and piglets), the authors further demonstrated that offspring's growth outcomes were improved by adding sialylated HMOs to their diet (87). These findings are complemented by studies looking at the effects of seasonality on breast milk composition and child health outcomes in rural communities. In the Gambia, for example, an increase in maternal energy intake during dry seasons (when food is plentiful and disease burden is low) resulted in significantly increased breast milk HMO production and was associated with decreased infant morbidity and improved infant weight- and height-for-age (88). Along with these outcomes, maternal malnutrition has also been associated with prenatal complications, preterm birth, low birth weight and chronic diseases in the later life of the infant (59, 89).

In contrast to maternal undernutrition, the effects of child undernutrition on the infant gut microbiota and health outcomes have been well-studied. In general, *Proteobacteria* (particularly *E. coli*) and *Streptococcus* are increased in the gut microbiota of undernourished children, while *Bacteroidetes* and anaerobic *Firmicutes* are decreased [reviewed in (90)]. These changes are associated with the possible inefficient transmission of health-related maternal gut microbiota. Accordingly, childhood undernutrition is quite possibly associated with the combined insult of the presence of highly pathogenic microbes and microbial deficiency in the guts of undernourished children (90). The plausibility of this is suggested by studies performed on children with severe acute malnutrition (SAM) caused by deficient breastfeeding and food and water insecurity (90). For example a longitudinal comparative study of the fecal samples of Malawian twin pairs discordant for SAM demonstrated that the gut microbiota of the twin with SAM was immature in comparison with the gut microbiota of their respective healthy twin control (91). Using gnotobiotic mice, the authors showed that the gastrointestinal tracts of mice containing the SAM microbiota were comprised of higher proportions of *Bilophila wadsworthia* and *Clostridium innocuum* in comparison to healthy controls; species that have been associated with inflammatory bowel disease and immune-compromised individuals, respectively (91–95). These findings suggest that these microbes are possibly associated with malnourishment on a more global scale.

In another study, children with SAM were provided with ready-to-use therapeutic food (RUTF; made from peanut paste, sugar, vegetable oil, and milk fortified with vitamins and minerals) and administered broad-spectrum oral antimicrobial agents (96). With this combination, children with SAM exhibited reduced mortality and improved recovery (94–96). These findings suggest that the antibiotic treatment may have targeted potential pathobionts in the gut, thereby facilitating the colonization of beneficial bacteria in accordance with the specific diet of the individual. This is further validated by a study that showed that chronic malnourishment in children (aged 2–5 years) was associated with an overgrowth of oral bacteria in the gut (97).

Aside from the effects of child malnutrition on the infant gut microbiota, child malnutrition in general can also impact the health outcomes of children, and has been associated with infection, death, and poor outcomes regarding growth, cognition, school-readiness, school performance, and earning potential in later life (59).

Given that hidden hunger was only recently described, and is complex to analyze, the specific effects of maternal and child hidden hunger on the microbiota and child health outcomes have not yet been well-studied. It has been noted, however, that maternal hidden hunger during pregnancy is associated with maternal morbidity and mortality, neural tube defects and impaired cognitive development in newborns, preterm births, and low birth weight (59). Child hidden hunger, on the other hand, is associated with poor outcomes regarding growth, development, immunity, tissue development, and health (59).

## Medication Use, the Gut Microbiota, and Health Outcomes

### The Effects of Medication Use on the Gut Microbiota and Health Outcomes

The effects of medication use on the gut microbiota have been extensively described in adults. It is known that antibiotics and some non-antibiotic drugs (such as proton pump inhibitors) can target the native bacteria of the gut and impair the microbial ecology of the gut, thereby leaving the gut susceptible to colonization by extrinsic commensal bacteria (such as microbes from the mouth) and enteric pathogens (3, 25, 98–100). Changes to gut microbiota composition due to antibiotic drugs can persist well after antibiotic administration, though in some cases the gut microbiota can recover within 1 month (101–103). Medication use also causes intestinal dysbiosis, which renders the gut susceptible to infections, dysregulates metabolism, and immune system homeostasis, and can lead to long-term metabolic effects, such as obesity (104–107). Moreover, continued exposure to both antibiotics and some non-antibiotic drugs can increase the presence of antibiotic-resistant genes in the gut microbiota (3, 25, 99, 105, 108).

Of the studies that have been performed during prenatal and early life, most concern the effects of antibiotic use. Maternal antibiotic use during the prenatal period, for example, has been shown to impact both the maternal gut microbiota and the breast milk microbiota, and is associated with an increased risk of childhood obesity (109, 110). Similarly, studies performed on the effects of antibiotics on children demonstrated that antibiotic use was associated with a less diverse gut microbiota at both the species- and strain-level, with a reduction in *Clostridiales* and an increase in antibiotic-resistant bacteria [Table 1; (9, 34)]. The negative effects of early life antibiotic use are so evident that even the positive effects of long breastfeeding duration on the infant gut microbiota and childhood BMI is reduced by early life exposure to antibiotics (111).

### The Effects of Urbanization on Medication Use

#### The Effects of Urbanization on Medical Practices and Behaviors

Medical practices and behaviors, such as antibiotic overuse or misuse and traditional and complementary medication use, have been shown to differ across urbanization gradients and can result in early life medication exposure. Distal factors associated with urbanization that impact medical practices and behaviors include policies and regulatory systems, the medication environment (i.e., accessibility, availability, and affordability of medications) and cultural beliefs (Figure 2).

Globally, and across urbanization gradients, only half of antibiotics are used correctly (112). This, as well as the misuse of antibiotics, can be attributed to unstandardized, and often poor, policies, and regulatory systems (41). An example of the latter is the easy acquisition of antibiotics from local shops or over the internet without prescription in some geographical locations (113, 114). Moreover, this relationship can also be observed in animal husbandry and agriculture (41). In the USA (an urban and high-income country, Box 1), for example, 80% of the total annual antimicrobial consumption is attributed to the livestock sector (112). Given that humans consume livestock, overuse or misuse of antimicrobials in livestock is also a concern and could be an additional route of early life exposure to antibiotics (115).

Contrary to popular belief, the use of traditional and complementary medication (TCM) is also widely common globally (116). Different factors can contribute to TCM use, but it often complements conventional medication use in more urban countries, whereas less urban countries are more dependent on TCM as a form of primary healthcare (116). The latter is due to the easier access of TCM and the cultural beliefs/acceptance of TCM in these regions (116, 117).

While commonly used to treat chronic medications, there is increasing evidence for the use of maternal TCM (particularly herbal medicine) during pregnancy, childbirth and postpartum, including during breastfeeding (117–121). Studies regarding the effects of TCM use on breast milk components, the breast milk microbiota, and the gut microbiota are lacking. However, an article by An et al. (122) reasoned that herbal medications can affect the gut microbiota in one of two ways. The first is via metabolism of herbal medication by the gut microbiota. The second is by regulating the composition and metabolites of the gut microbiota (122). An example of the latter is that certain herbal medications can act as probiotics and promote the growth of beneficial gut microbiota (122). The benefits and limitations of probiotic use, and its effects on the gut microbiota, have already been extensively [reviewed in (123)]. Another study, also investigating the effects of herbal medication on the gut microbiota, showed that some herbal medication supplementation can promote gut bacteria associated with positive health outcomes and reduce the relative abundance of potential pathogens (124). Though the reported outcomes of some TCM use on the gut microbiota seem promising, it is important to acknowledge that more research on TCM needs to be performed, particularly relating to drug–drug interactions and possible adverse side-effects (116). Given the increased statistics of TCM use worldwide, and its use during pregnancy, childbirth, and postpartum (especially during breastfeeding), more knowledge needs to be obtained about the effects of using various kinds of TCM on the gut microbiota and health outcomes of infants.

#### The Effects of Urbanization on Medication Use Associated With Maternal and Child Disease

The prevalence of communicable diseases (CDs) and non-communicable diseases (NCDs) differ across urbanization gradients and associated income groups (Boxes 1, 2). In 2016, for example, 50% of all deaths in low-income countries were due to CDs, nutritional deficiencies and maternal complications during pregnancy and childbirth, while only 7% of deaths in high-income countries were due to these causes (125). In contrast, of the 71% of deaths caused by NCDs, 88% occurred in high-income countries (125). Along with these statistics, low– and middle–income countries are experiencing a rise in NCDs, resulting in a double burden of CDs and NCDs in these countries (5, 59, 125). Accordingly, the gut microbiota and health outcomes of infants can be differentially influenced by medication use associated with maternal and child disease across urbanization gradients.

The effects of NCDs on the gut microbiota (and vice versa) and their health outcomes have been well-studied (4), while the effects of CDs have been understudied. This narrative review will highlight some of the few studies that have been performed on the effects of maternal and child CDs and associated medication use on the gut microbiota and health outcomes of infants. As medication use is closely linked with disease, it can be difficult to distinguish the effects of medication use and disease on the gut microbiota.

One example of a maternal medication that is used as a result of disease is antiretroviral therapy (ART), which is used to combat HIV. Although HIV has been relatively well-managed globally, it is still a major health concern in sub-Saharan Africa [which is considered relatively less urban, Box 1; (126)]. While there have been studies on the effects of HIV on breast milk composition [(127); reviewed in (110)], the combined effects of HIV and ART on the maternal gut microbiota during pregnancy have been little studied. There is some evidence, however, of the effects of HIV and ART exposure during pregnancy on the gut microbiota and health outcomes of infants.

Due to preventative treatment administered to HIV-positive mothers during pregnancy, per year more than 1 million infants are born HIV exposed but uninfected with HIV (HEU) to HIV-positive mothers worldwide. The mortality rates of these infants are twice that of infants not exposed to HIV from the same environment (128–130). In addition to high mortality rates, HEU infants also experience decreased growth, differences in metabolism and immune system development, and increased susceptibility to infections (130–135). Moreover, the gut microbiota composition of HEU infants differs from that of infants unexposed to HIV, and the gut bacterial communities of HEU infants are also less mature—a characteristic generally observed in HIV-positive individuals (136). As maternal gut microbes can be transmitted to the infant during delivery, it may be the impact of HIV and ART on the maternal gut microbiota, along with changes to the breast milk composition (as mothers with HIV are still advised to breast feed), that contributes to the differences observed in HEU babies. Moreover, HEU babies are placed on prophylactic ART treatment for 4–6 weeks following birth, and this too could have a major impact on the gut microbiota and health outcomes of these infants (137). Studies focused on the effects of ART in general have demonstrated that ART prevents restoration of gut symbiosis, thereby potentially facilitating gut dysbiosis (138–143).

Diarrhea is the second leading cause of death in children under 5 years of age, leading to an estimated 1.5 million deaths each year (144, 145). The combined insult of diarrhea and medication use (and often also severe malnutrition) can have enormous negative effects on the gut microbiota and health outcome of infants and children (50, 59, 90). For example the combined treatment of antibiotics and iron fortification supplements that is generally provided to sub-Saharan African infants to treat or prevent iron-deficiency anemia can contribute to childhood diarrheal prevalence (146, 147). Moreover, a small study by Paganini et al. (147) demonstrated that this combined treatment resulted in a decreased abundance of *Bifidobacterium* (bacteria associated with a healthy gut microbiota) and no decrease in pathogenic *E. coli* (one of the bacterial species known to cause diarrhea) in the infant gut. These findings highlight the importance of exhibiting caution when providing infants with antibiotic and iron fortification supplements when they are suffering from and being treated for diarrheal infections. These results further indicate the possibility of modulating the gut microbiota to potentially facilitate positive health outcomes in the event that there is no other option than to provide combined treatment. In this case, it would be interesting to determine whether the addition of *Bifidobacterium* probiotics (at the strain level) could improve outcomes with the combined antibiotic and iron fortification supplementation treatment.

Interestingly, the relationship between the childhood gut microbiota and infections can also determine the efficacy of certain childhood medications, and thereby the health outcomes of infected children. An example of this is the rotavirus vaccine (RVV), which is used to treat rotavirus infection, one of the key causes of diarrhea-related deaths. A pilot study by Harris et al. (148) hypothesized and demonstrated that low RVV efficacy was correlated with microbiota composition in a cohort of Pakistani infants. The gut microbiota of infants who responded to the RVV had an increased relative abundance of broadly categorized pathogenic bacteria (e.g., *Clostridium cluster XI* and *Proteobacteria*) in comparison to non-responders. Moreover, higher RVV efficacy was generally observed in a Dutch infant cohort of RVV-responders, demonstrating higher RVV efficacy in infants from high-income countries (148). This could, in part, be explained by differences in breast milk HMO composition across geographical regions and urbanization gradients. Indeed, a study by Ramani et al. (149) demonstrated that a particular geographically distinct neonatal rotavirus strain in India was increased in an *in vitro* model in response to particular HMO constituents. These findings highlight the importance of understanding how the effects of urbanization across geographical locations can impact HMO composition and, as such, influence medication response in children with disease.

## Future Perspectives and Concluding Remarks

This narrative review highlights some of the many factors associated with urbanization that differentially affect delivery mode, nutrition, and medication use, which in turn differentially affect the gut microbiota and health outcomes of infants across urbanization gradients. We have shown that urbanization contributes to: C-section overuse in urban communities, poor infant feeding practices and behaviors across urbanization gradients, different degrees and forms of malnutrition across urbanization gradients, and early life medication exposure due to both medical practices and behaviors, and distinct disease burdens across urbanization gradients. All of these relationships have varying effects on the gut microbiota and health outcomes of infants.

Taking these relationships and their effects into consideration, we conclude and propose the following:

- The overuse of C-section deliveries, particularly in urban areas, highlights the need for global, standardized, and ethical C-section delivery recommendations/guidelines, and maternal education on delivery rights. Interventions to optimize C-section use have been well-reviewed by the “Optimizing Cesarean Section Use” Lancet series (48).
- Exclusive breastfeeding is highly recommended during early life due to its clear benefits (150). However, for these recommendations to be realized, factors associated with urbanization that contribute to early breastfeeding cessation or non-exclusive breastfeeding need to be taken into account. Our narrative review highlights the important need for:
  ◦ Governmental support for breastfeeding practices (particularly in public and workplace environments) to normalize breastfeeding in our societies, to prevent the negative effects of unpaid and/or little parental leave, and to encourage mothers, particularly working mothers, to exclusively breastfeed.
  ◦ Societal education and awareness about proper exclusive breastfeeding practices and their benefits (from public to workforce and healthcare systems).
- The various examples of maternal and child nutrition and malnutrition in this review highlight the important need to improve food environments, food practices, and nutrition education across urbanization gradients. This has already been well and extensively reviewed in a recent UNICEF report (59). Moreover, we showed in this review the important interaction between diet, the gut microbiota and health outcomes, and how essential it is to monitor the gut microbiota, for example to determine the effects of ready-to-use therapeutic foods (and in some cases antibiotic use) on the gut microbiota and health outcomes of children with SAM.
- Medical practices and behaviors across urbanization gradients contribute to early life medication exposure as a result of poor policies and regulatory systems, medication environments, and cultural beliefs. This highlights the need to standardize policies and regulatory systems (for both human and agricultural medication use), to improve medication environments (by improving accessibility, availability and affordability of high-quality, regulated medications) and to improve our understanding of how cultural beliefs can contribute to these practices and behaviors. It also highlights the importance of educating the public of the effects of medication use and to only use medication if absolutely necessary.
- Medication use associated with maternal and child disease differs across urbanization gradients, with a lack of studies performed on the effects of CDs on the gut microbiota and health outcomes of infants. CDs and associated medication use not only differentially impact the gut microbiota and health outcomes of infants and children, but they can also contribute to reducing medication efficacy. Accordingly, knowledge of these interactions can facilitate targeted modulation of the gut microbiota to facilitate positive health outcomes for infected mothers and children because, more often than not, medication use associated with disease is required to fight the disease. Moreover, these findings highlight the importance of taking these factors into consideration when implementing applications and policies to improve the health outcomes of mothers and children with disease.

The factors covered in this narrative review are numerous, but not exhaustive. Other factors to be considered in future studies include various genetic, epigenetic, and environmental factors such as infant sex, parental race/ethnicity, parental diseases, smoking, pet exposure, stress/depression, pollution, climate change, marginalized communities (such as refugees), and cultural practices (such as the Chinese “sitting in period”) (12, 59, 151). Irrespective, based on the examples provided in this narrative review, the differential effects of urbanization on the gut microbiota and health outcomes of infants are clear.

To further expand our understanding of how the microbiota impacts health and can be modified to improve health in a global context, microbiota studies should be continued in both urban and rural populations, and factors associated with urbanization that can contribute to maternal and infant health outcomes should be taken into consideration. By deepening our understanding of these relationships, and by increasing studies across urbanization gradients, we can identify and implement new biological and social approaches to prevent and treat disease and promote positive maternal and infant/childhood health world-wide.

## Author Contributions

SB and TS performed the literature search and wrote the manuscript. SR, MK, and AZ contributed to writing and critical editing of the paper. All authors contributed to manuscript revision, read, and approved the submitted version.

## Conflict of Interest

The authors declare that the research was conducted in the absence of any commercial or financial relationships that could be construed as a potential conflict of interest.
