# Supplementary material for: The Effects of Urbanization on the Infant Gut Microbiota and Health Outcomes
Source: Front Pediatr. 2020 Jul 29;8:408. doi: 10.3389/fped.2020.00408 (PMC7438894; doi:10.3389/fped.2020.00408)
Supplement: Supplementary file 1 [file Table_1.DOCX]

Supplementary Material

# Search Strategy and Criteria for Narrative Review

This review was written as a narrative review. We used the following procedure to identify relevant articles:

First we focused on the well-studied factors known to influence the infant gut microbiota, e.g. delivery mode, nutrition and medication use, using the following search terms:

[(“infant*” OR “baby” OR “child”)] AND [(“microbiome” OR “microbiota” OR “microbial communities”)] AND [(“gut” OR “intestinal” OR “stool” OR “meconium”)] AND [factor]

where factor regards:

- delivery mode terms: [(“delivery mode” OR "caesarean delivery" OR “c-section” OR “caesarean” OR “vaginal” OR "vaginal delivery" OR "mode of birth” OR "mode of delivery”)]
- nutrition terms [(“nutrition” OR “breast feeding” OR “breastfed” OR “formula” OR “mixed feeding” OR “food” OR “solid food”)]
- medication use terms [(“antibiotic*” OR “non-antibiotic*” OR “medication” OR “traditional medication” OR “complementary medication”)]

As our primary interest was to highlight the differences in these core relationships associated with urbanization, we referred to the World Bank and United Nation resources to define “urbanization”. With this information, together with resources from the WHO and UNICEF, we included the following search terms:

[(“urban” OR “westernized” OR “western” OR “rural” OR “traditional”] for each factor (as indicated in point 1 above) and also included search terms for specific health outcomes where necessary, e.g. [(“malnutrition” OR “overweight” OR “obese” OR “underweight” OR “stunted” OR “wasting” OR “severe acute malnutrition”)] etc.

We searched the PubMed database using the above defined search terms for English publications published within the last 5 years or an extended period if insufficient information, with a focus on human-related studies. Abstracts were sorted according to their relevance for each topic outlined in the narrative review, and the study designs were critically evaluated before selection. We also reviewed the references of the selected studies for possible additional publications and included all eligible publications.
